# MAST1 modulates neuronal differentiation and cell cycle exit via P27 in neuroblastoma cells

**DOI:** 10.1002/2211-5463.12860

**Published:** 2020-04-29

**Authors:** Tianrui Jing, Jing Ma, Huanqiang Zhao, Jin Zhang, Nan Jiang, Duan Ma

**Affiliations:** ^1^ Key Laboratory of Metabolism and Molecular Medicine Ministry of Education Department of Biochemistry and Molecular Biology School of Basic Medical Sciences & Institutes of Biomedical Sciences Shanghai Medical College Fudan University Shanghai China; ^2^ Department of Facial Plastic and Reconstructive Surgery ENT Institute Eye & ENT Hospital Fudan University Shanghai China; ^3^ Obstetrics and Gynecology Hospital Fudan University Shanghai China; ^4^ Children's Hospital Fudan University Shanghai China

**Keywords:** cell cycle, MAST1, neuronal differentiation, P27, SH‐SY5Y

## Abstract

Although 19p13.13 microdeletion syndrome has been consistently associated with intellectual disability, overgrowth, and macrocephaly, the underlying mechanisms remain unclear. MAST1, a member of the microtubule‐associated serine/threonine kinase family, has been suggested as a potential candidate gene responsible for neurologic abnormalities in 19p13.13 microdeletion syndrome, but its role in nervous system development remains to be elucidated. Here, we investigated how MAST1 contributes to neuronal development. We report that MAST1 is upregulated during neuronal differentiation of the human neuroblastoma cell line, SH‐SY5Y. Inhibition of MAST1 expression by RNA interference attenuated neuronal differentiation of SH‐SY5Y cells. Cell cycle analyses revealed that MAST1‐depleted cells did not undergo cell cycle arrest after RA treatment. Consistent with this observation, the number of EdU‐positive cells significantly increased in MAST1 knockdown cells. Intriguingly, levels of P27, a cyclin‐dependent kinase inhibitor, were also increased during neuronal differentiation, and MAST1 knockdown reduced the expression of P27. Moreover, reduced neuronal differentiation caused by MAST1 depletion was rescued partially by P27 overexpression in SH‐SY5Y cells. Collectively, these results suggest that MAST1 influences nervous system development by affecting neuronal differentiation through P27.

## Graphical abstract

During neurogenesis, neuronal differentiation and cell cycle are closely coordinated. MAST1, a member of the microtubule‐associated serine/threonine kinase family, contributes to neuronal differentiation and cell cycle exit by regulating P27 in neuroblastoma cells.

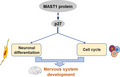

**Abbreviations**
CACNA1A: calcium voltage‐gated channel subunit alpha1 A
CKIs: cyclin‐dependent kinase inhibitors
EdU: 5‐ethynyl‐2‐deoxyuridine
MAP2: microtubule‐associated protein 2
MAST1: microtubule‐associated serine/threonine kinase 1
NFIX: nuclear factor I X
PDZ: postsynaptic density protein 95/disks large/zona occludens‐1 domain
PFA: paraformaldehyde
RA: all‐trans‐retinoic acid
SD: standard deviation
shRNAs: short hairpin RNAs

A novel microdeletion of 19p13.13 is consistently associated with moderate intellectual disability, developmental delay, abnormalities of brain structure, macrocephaly, and overgrowth [1, 2]. The locus of the human *MAST1* gene is present in the common deletion region and is considered to be one of the candidate genes of 19p13.13 microdeletion syndrome [3]. MAST1 is characterized by a serine/threonine kinase domain and a postsynaptic density protein 95/disks large/zona occludens‐1 domain (PDZ) [4], which gives MAST1 the ability to scaffold its own kinase activity. The *MAST1* gene has been shown to be expressed in many brain areas including the hippocampus, cerebellum, 3rd ventricle, and cerebral cortex [4]. In the nervous system, MAST1 plays a critical role through localization within the utrophin/dystrophin‐associated complex, which is found within the postsynaptic region of the neuromuscular junction and central synapses [5]. The sequence C‐terminal of the PDZ domain is highly variable in MAST1, which affects its subcellular localization within neurons [6]. Previous studies revealed that MAST1 was a novel candidate gene in cerebral palsy and intellectual disability gene [7, 8] and was associated with Alzheimer's disease [9]. These observations indicated MAST1 may have a function in neuronal development and may be a new potential biomarker in neuronal development disorders. However, evidence has not been forthcoming.

During neurogenesis, neuronal differentiation progression and cell cycle regulation are closely coordinated [10, 11]. To start terminal differentiation, neuronal stem cells must exit the cell cycle, indicating the existence of crosstalk signal pathways between neuronal differentiation and cell cycle. However, the relationship between molecule mechanisms associated with cell cycle regulation and neuronal differentiation progression remains largely unknown. Cyclin‐dependent kinase inhibitors (CKIs) play an important role in regulating neuronal differentiation and the cell cycle [12, 13, 14, 15]. CKIs comprise two families: CDK‐interacting/kinase inhibition protein (Cip/Kip; P21, P27, and P57) and inhibitors of CDK4 (P15, P16, P18, and P19). Notably, P27 is particularly important for neuronal differentiation and neurogenesis [16, 17]. P27 promotes cell cycle exit and neuronal differentiation both *in vivo* [18] and *in vitro* studies [19].

In our study, we observed striking increases in MAST1 expression during neuronal differentiation. Reducing MAST1 expression impaired SH‐SY5Y neuronal differentiation and interfered in cell cycle exit. We further explored the mechanisms and found that P27 decreased in MAST1 knockdown cells. Moreover, P27 re‐expression partially rescued the effect of MAST1 knockdown on neuronal differentiation. Taken together, the data reveal that P27 meditates MAST1 function in neuronal differentiation.

## Methods and materials

### Antibodies

The following antibodies were used for immunofluorescence and/or western blot analyses. Antibodies against MAP2, P27, P21, and P57 were purchased from Cell Signaling Technology (Danvers, MA, USA). Antibodies against β‐actin were purchased from Proteintech (Wuhan, China). Antibody against GAPDH and MAST1 was purchased from Sigma‐Aldrich (St. Louis, MO, USA) and Novus Biologicals (Centennial, CO, USA), respectively.

### Immunofluorescence

Cells were washed three times with PBS and fixed for 30 min at room temperature in 4% paraformaldehyde (PFA). Cells were permeabilized with 0.5% Triton X‐100 in PBS for 20 min and then blocked with 5% BSA for 1 h. Antibodies were incubated for 12 h at 4 °C. Cells were washed three times with PBS and then incubated with fluorescence‐conjugated secondary antibodies and DAPI at room temperature for 2 h. Coverslips were mounted and sealed on slides. Images were taken using fluorescence microscopy (Thermo Fisher Scientific, Waltham, MA, USA).

### Cell culture and differentiation

Human neuroblastoma SH‐SY5Y (Cell Bank of Type Culture Collection of the Chinese Academy of Sciences, Shanghai, China) and mouse neuroblastoma N_2_a (Cell Bank of Type Culture Collection of the Chinese Academy of Sciences) were maintained in Dulbecco's modified Eagle medium: Nutrient Mixture F‐12 (DMEM/F12) containing 10% FBS in a humidified atmosphere at 37 °C. To induce differentiation, SH‐SY5Y and N_2_a cells were incubated in DMEM/F12 with 1% FBS and 10 μm all‐trans‐retinoic acid (RA; Sigma‐Aldrich) for 8 days. The neurite outgrowth of SH‐SY5Y cells was counted by using Neuron J plugin for image j (NIH, Bethesda, MD, USA). The mouse embryonic carcinoma cell line P19 (Cell Bank of Type Culture Collection of the Chinese Academy of Sciences) was maintained in a‐MEM medium supplemented with 10% FBS at 37 °C. To induce P19 cell differentiation, cells were allowed to aggregate in bacterial‐grade Petri dishes in the presence of 1 μm RA. After 4 days of aggregation, cells were dissociated into single cells and were plated in a poly‐l‐lysine‐coated tissue culture dish in Neurobasal‐A medium (Invitrogen, Waltham, CA, USA) with B27 (Invitrogen) supplement. Cells were allowed to attach for 24 h and then were treated with 10 μm Ara‐C for 24 h to suppress growth of non‐neuronal cells. All cell culture dishes and culture plates were purchased from Hangzhou Xinyou Biotechnology Co., Ltd, Hangzhou, China.

### RNA isolation and real‐time quantitative PCR

Total RNA was isolated using the TRIzol reagent (Invitrogen) following the manufacturer's instructions. Each sample (1 μg) was used to synthesize cDNA using the PrimeScript™ RT Reagent Kit (Takara, Kusatsu, Shiga, Japan). Real‐time PCR was performed using SYBR^®^ Premix Ex Taq™ II (Takara). PCRs were performed with the following PCR cycles: 95 °C for 10 min; 40 cycles: 95 °C for 10 s, 60 °C for 30 s, and 72 °C for 30 s; and melt curve: 95 °C for 15 s, 60 °C for 1 min, and 95 °C for 15 s using an Eco™ quantitative PCR system (Illumina, San Diego, CA, USA). The following primers were used: MAST1, 5′‐TCTCTGGACCGCGCTTTCTA‐3′ and 5′‐TGAGGCTTTTCCGATTACTGGT‐3′; β‐ACTIN, 5′‐AGAGCTACGAGCTGCCTGAC‐3′ and 5′‐AGCACTGTGTTGGCGTACAG‐3′.

### Western blot analysis

Cells were washed three times with PBS and lysed with radioimmunoprecipitation assay buffer containing protease inhibitor cocktail (Roche Diagnostics, East Sussex, UK) for 30 min at 4 °C. The supernatant was collected after centrifugation at 12 000 ***g*** for 15 min at 4 °C. Protein concentrations were determined using the Bradford Protein Assay Kit (Beyotime Biotechnology, Shanghai, China). Equal amounts of protein were separated by an appropriate concentration of polyacrylamide gel electrophoresis and transblotted onto polyvinylidene difluoride–nitrocellulose filters. Membranes were blocked with 8% nonfat milk for 1 h at room temperature and then incubated with primarily antibodies at 4 °C overnight and secondary antibodies at room temperature for 2 h.

### Lentiviral vector production and infection

Short hairpin RNAs (shRNAs) included MAST1 shRNA, which targets the human *MAST1* transcript sequence 5′‐CCACTTCCTCTCCAAACACTT‐3′; P27 shRNA, which targets the human *P27* transcript sequence 5′‐GTCAAACGTGCGAGTGTCTAA‐3′; and the control (pGreen‐CMV‐puro‐vector) plasmid. The shRNAs included Mast1 shRNAs, which targets the mouse *Mast1* transcript sequences, shmMast1‐1, 5′‐GCACAGCTTCTAATATCTAGC‐3′; shmMast1‐2, 5′‐GCAAGGTGTACAGCAGTATGG‐3′; shmMast1‐3, 5′ GCACAGACTCCAAGGGATTGA‐3′; and the control (pLKO.1‐puro‐vector) plasmid. Viral supernatant was collected 48–72 h post‐transfection and concentrated through ultracentrifugation. Cells were infected twice with viruses in the presence of 8 μg·mL^−1^ polybrene (Genomiditech, Shanghai, China). After 48 h, cells were selected and maintained in medium containing 1 μg·mL^−1^ puromycin. To overexpress P27, P27 cDNA was subcloned into the pLVX‐IRES‐Hyg vector, transfected into cells, and selected with 300 μg·mL^−1^ of hygromycin.

### Cell cycle analysis

Cells were trypsinized, washed three times with PBS, and fixed with 70% ethanol for 12 h at 4 °C. After washing twice with PBS, the fixed cells were resuspended in PI/RNase staining buffer (BD Biosciences, San Jose, CA, USA) for 10 min in the dark at 37 °C before analysis. Cell cycle analysis was performed using a FACSCalibur flow cytometer (BD Biosciences).

### 5‐ethynyl‐2‐deoxyuridine incorporation immunofluorescence

5‐ethynyl‐2‐deoxyuridine (EdU; RiboBio, Guangzhou, China) at 50 μm was added to the media and incubated for 2 h before fixation. Cell was washed three times with PBS and fixed for 30 min at temperature in 4% PFA and subsequent EdU detection per the manufacturer's protocol. Cells were permeabilized with 2 mg·mL^−1^ glycine for 5 min and then were incubated with 0.5% Triton X‐100 in PBS for 10 min on shaker. Coverslips were mounted on slides, and cells were imaged using fluorescence microscopy. Quantification of EdU‐positive cells was carried out using the image j software (NIH). The percentage of EdU‐positive for each field of view captured was recorded and analyzed.

### Statistical analysis

Data were established as the mean ± the standard deviation (SD). Comparisons between groups were analyzed using a two‐tailed unpaired Student's *t*‐test or by one‐way ANOVA followed by a LSD *post hoc* test, as required. A *P* < 0.05 was regarded as statistically significant.

## Results

### MAST1 expression increases during SH‐SY5Y cell neuronal differentiation

**Fig. 1:**
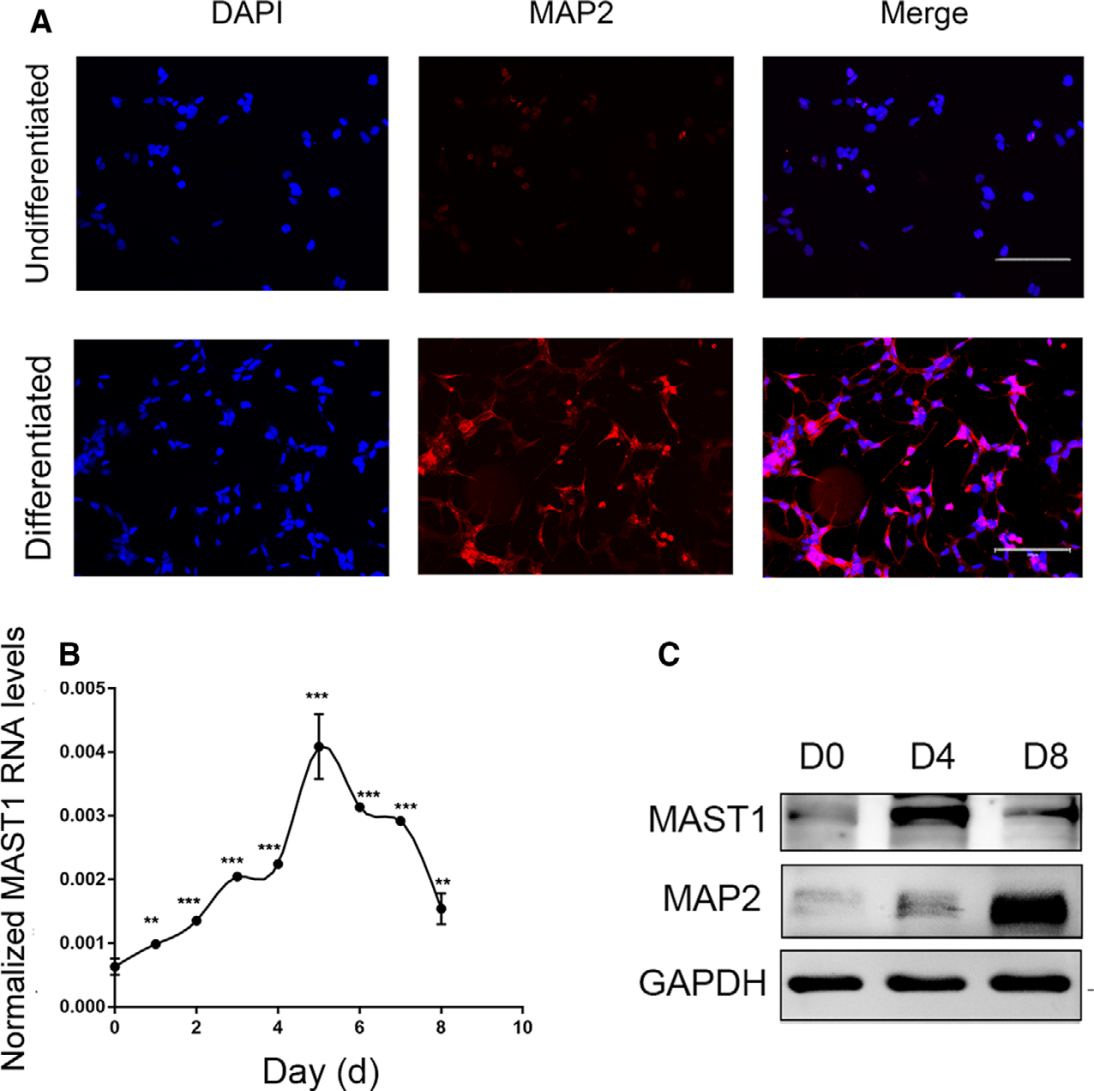
Induced expression of MAST1 during SH‐SY5Y cell neuronal differentiation. (A) Monolayer undifferentiated and neuronally differentiated SH‐SY5Y cells which had cultured in the absence or presence of 10 μm RA for 8 days were immunostained with MAP2 (red fluorescence) antibody and DAPI (blue fluorescence). (B) RT–PCR analysis of MAST1 mRNA levels, normalized to β‐ACTIN, from different days of SH‐SY5Y cell neuronal differentiation. (C) Western blot analysis of protein expressions of MAST1 and the neuronal markers MAP2, from different phases of SH‐SY5Y cell neuronal differentiation. D, day. Scale bars, 200 μm. Data are depicted as means ± SD of at least three independent experiments. Statistical analysis was performed using one‐way ANOVA followed by the LSD *post hoc* test. **P < 0.01, ***P < 0.001 versus D0.

The human SH‐SY5Y neuroblastoma cell line is a well‐established neuronal precursor line that can be induced by RA to differentiate into neurons for studying neuronal differentiation [20, 21]. With RA treatment for 4–8 days, a majority of the SH‐SY5Y cells gradually become postmitotic neurons, characterized by longer neurite outgrowths [22]. To evaluate whether RA effectively induced neuronal differentiation, we used MAP2, a neuron marker enriched in dendrites, to monitor the neuronal differentiation of SH‐SY5Y cells [23]. As shown in Fig. 1A, undifferentiated SH‐SY5Y cells expressed a low level of MAP2. Conversely, differentiated SH‐SY5Y cells showed strong positive staining with the anti‐MAP2 antibody. Besides MAP2, the neuron‐specific III neuron‐tubulin (TUBB3) was used to verify SH‐SY5Y neuronal differentiation. Western blot showed that the expression of TUBBB3 increased after RA treatment (Fig. S1). During SH‐SY5Y cell neuronal differentiation, RT–PCR showed the expression of MAST1 gradually increased from day 1, peaked at the fifth day, and decreased from day 6 to day 8 in the presence of RA (Fig. 1B). We also observed the protein expression of MAST1 in SH‐SY5Y, N_2_a, and P19 cells and found that the pattern of MAST1 protein expression was consistent with MAST1 mRNA expression (Fig. 1C, Fig. S2). Taken together, these data revealed that the expression of MAST1 increased during the neuronal differentiation of SH‐SY5Y cells and provided a precise time course of MAST1 gene expression.

### MAST1 inhibition by RNA interference attenuates neuronal differentiation of SH‐SY5Y cells

**Fig. 2:**
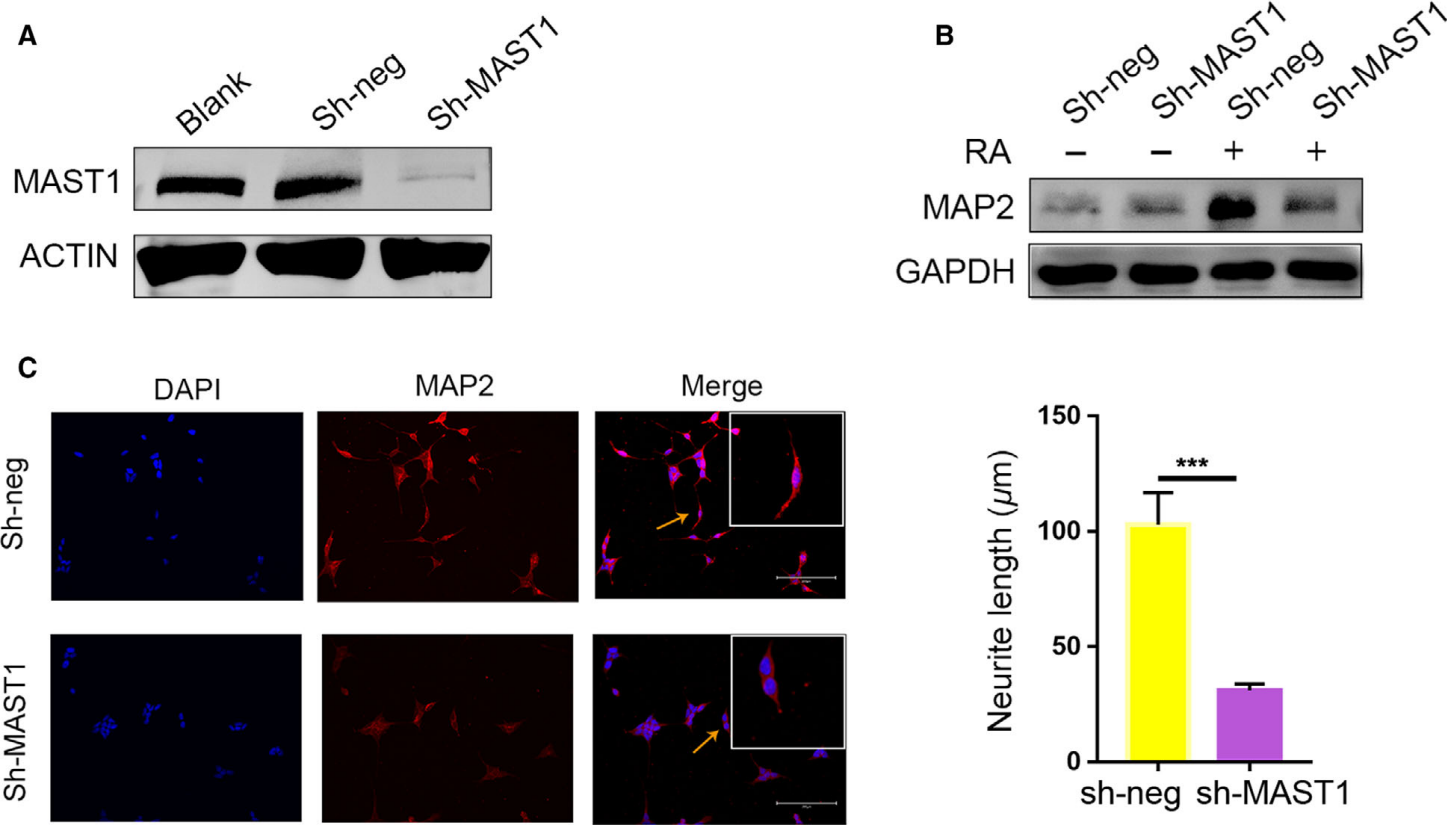
RNA interference targeting MAST1 impairs the neuronal differentiation of SH‐SY5Y cells. SH‐SY5Y cells were infected with sh‐MAST1 or control (sh‐neg) lentiviruses. (A) Western blot analysis of MAST1 protein expressions in noninfected, control (sh‐neg), and sh‐MAST1 lentiviruses infected SH‐SY5Y cells. (B) Western blot analysis of MAP2 protein expression in control (sh‐Neg), or sh‐MAST1 lentiviruses infected SH‐SY5Y cells, which were cultured in the absence or presence of RA for 8 days. (C) Immunostaining of neuronally differentiated SH‐SY5Y cells, which were cultured in the presence of RA for 8 days, with anti‐MAP2 antibody. The neurite length was calculated in differentiated SH‐SY5Y cells (arrows: representative neurite). Scale bars, 200 μm. Data are depicted as means ± SD of at least three independent experiments. ***P < 0.001, as determined by the two‐tailed unpaired Student's *t*‐test.

In order to clarify the function of MAST1 in neuronal differentiation, we used shRNA to silence MAST1 and sought to uncover the effect of MAST1 knockdown on SH‐SY5Y neuronal differentiation. We used virus sh‐MAST1 to target the MAST1 transcript region, which results in a dramatic decrease in the expression of MAST1 (Fig. 2A). On the contrary, the negative control had no effect on MAST1 expression. The neuronal marker MAP2 was used to monitor neuronal differentiation in MAST1 knockdown cells. High levels of MAP2 were found in the control (sh‐neg), whereas the expression of MAP2 was significantly reduced in MAST1 knockdown cells (Fig. 2B). For morphological analysis, we performed immunofluorescence staining using MAP2 and found an attenuated extension of neurite in MAST1 knockdown cells (Fig. 2C). We also found that the percentage of differentiated cells decreased in Mast1 knockdown N_2_a cells (Fig. S3). These results showed that MAST1 has a critical role in SH‐SY5Y neuronal differentiation.

### Cell cycle exit is impaired by suppressing expression of MAST1 in SH‐SY5Y cells

**Fig. 3:**
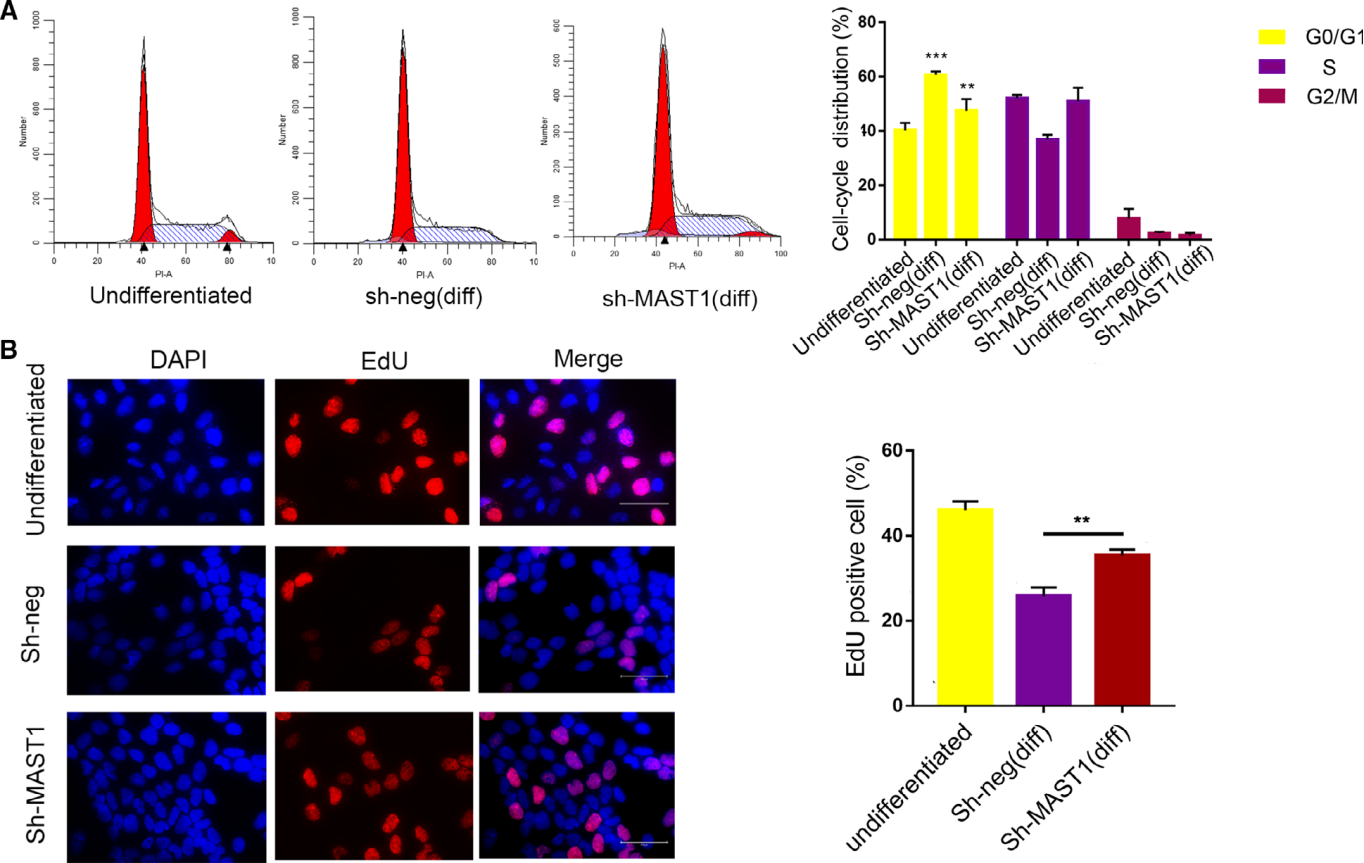
MAST1 knockdown affects the cell cycle exit in SH‐SY5Y cells. SH‐SY5Y cells were infected with a control (sh‐neg) or sh‐MAST1 lentiviruses. After 4 days of culture in the absence or presence of RA, phases of the cell cycle were detected by flow cytometry. (A) Flow cytometry data showed distribution of the cell cycle. Percentages of cells in each phase of the cell cycle are depicted in the graph on the right. (B) Representative photomicrograph of EdU immunostaining. SH‐SY5Y cells that were cultured in the presence of 10 μm RA for 4 days and incubated with EdU for 2 h before fixation. The percentage of EdU‐positive cells was determined. Scale bars, 100 μm. Data are depicted as means ± SD of at least three independent experiments. **P < 0.01, ***P < 0.001 versus undifferentiated, as determined by one‐way ANOVA followed by the LSD *post hoc* test.

Previous studies have shown that cell cycle exit represented the fundamental step to trigger cell differentiation [24, 25, 26]. Thus, we asked whether MAST1 modulates neuronal differentiation by promoting cell cycle arrest. To examine MAST1 function in the cell cycle during neuronal differentiation, flow cytometry was performed to test whether MAST1 knockdown attenuated cell cycle arrest in SH‐SY5Y cells after 4 days of RA treatment. Knockdown of MAST1 attenuated the decrease in the G0/G1 phase cell induced by RA treatment, indicating a disruption in cell cycle arrest (Fig. 3A). EdU assay was performed to detect cell proliferation, and the results were in agreement with flow cytometric analysis. The percentage of EdU‐positive cell increased in MAST1 knockdown cells (Fig. 3B). These data showed that MAST1 has an important role in cell cycle regulation, especially in blocking cells at the G0/G1 phase, and promoting neuronal differentiation.

### Downregulation of MAST1 inhibits P27 in SH‐SY5Y cells

**Fig. 4:**
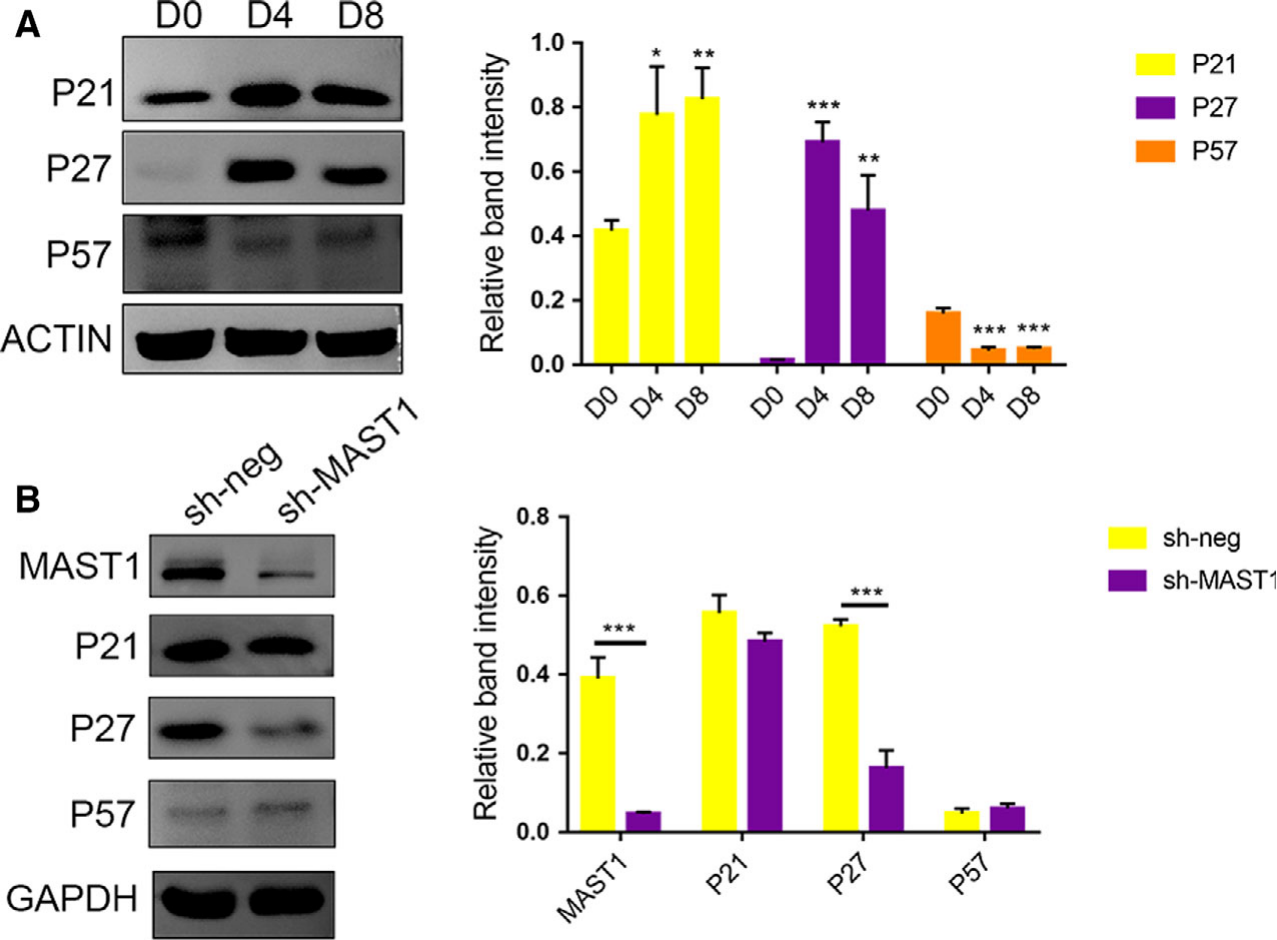
Expression patterns of Cip/Kip cyclin kinase inhibitors during SH‐SY5Y cell neuronal differentiation and the effect of MAST1 knockdown on P27. (A) Western blot analysis of P21, P27, and P57 protein levels from different phases of SH‐SY5Y cell neuronal differentiation. Western blots were quantified, and band intensities were presented relative to Day 0. (B) Western blot analysis of P21, P27, and P57 expressions in control (sh‐Neg), or sh‐MAST1 lentiviruses infected SH‐SY5Y cells, which were cultured in the presence of 10 μm RA for 4 days. Western blots were quantified, and band intensities were presented. Data are depicted as means ± SD of at least three independent experiments. *P < 0.05, **P < 0.01, and ***P < 0.001, as determined by one‐way ANOVA followed by the LSD *post hoc* test (A) and the two‐tailed unpaired Student's *t*‐test (B).

The Cip/Kip family of proteins, including P21, P27, and P57, inhibit a broad spectrum of cyclin‐dependent kinases and are especially important in regulating differentiation and proliferation during neurogenesis [16, 27]. Given the dual functions of MAST1 on the regulation of neuronal differentiation and cell cycle, we speculated that the Cip/Kip family meditated the function of MAST1 in neuronal differentiation. The expression patterns of P21, P27, and P57 were examined during SH‐SY5Y cell neuronal differentiation. P27 and P21 expression, similar to MAST1, dramatically increased during SH‐SY5Y cell neuronal differentiation, but P57 expression was downregulated (Fig. 4A). To assess this increase, we examined the levels of P21, P27, and P57 in MAST1 knockdown SH‐SY5Y cells. As expected, P27 was decreased in MAST1 knockdown SH‐SY5Y cells, while P21 and P57 remained unchanged (Fig. 4B). These results suggested that MAST1 regulates P27, and not P21 or P57.

### Re‐expression of P27 partially rescues neuronal differentiation in MAST1 knockdown cells

**Fig. 5:**
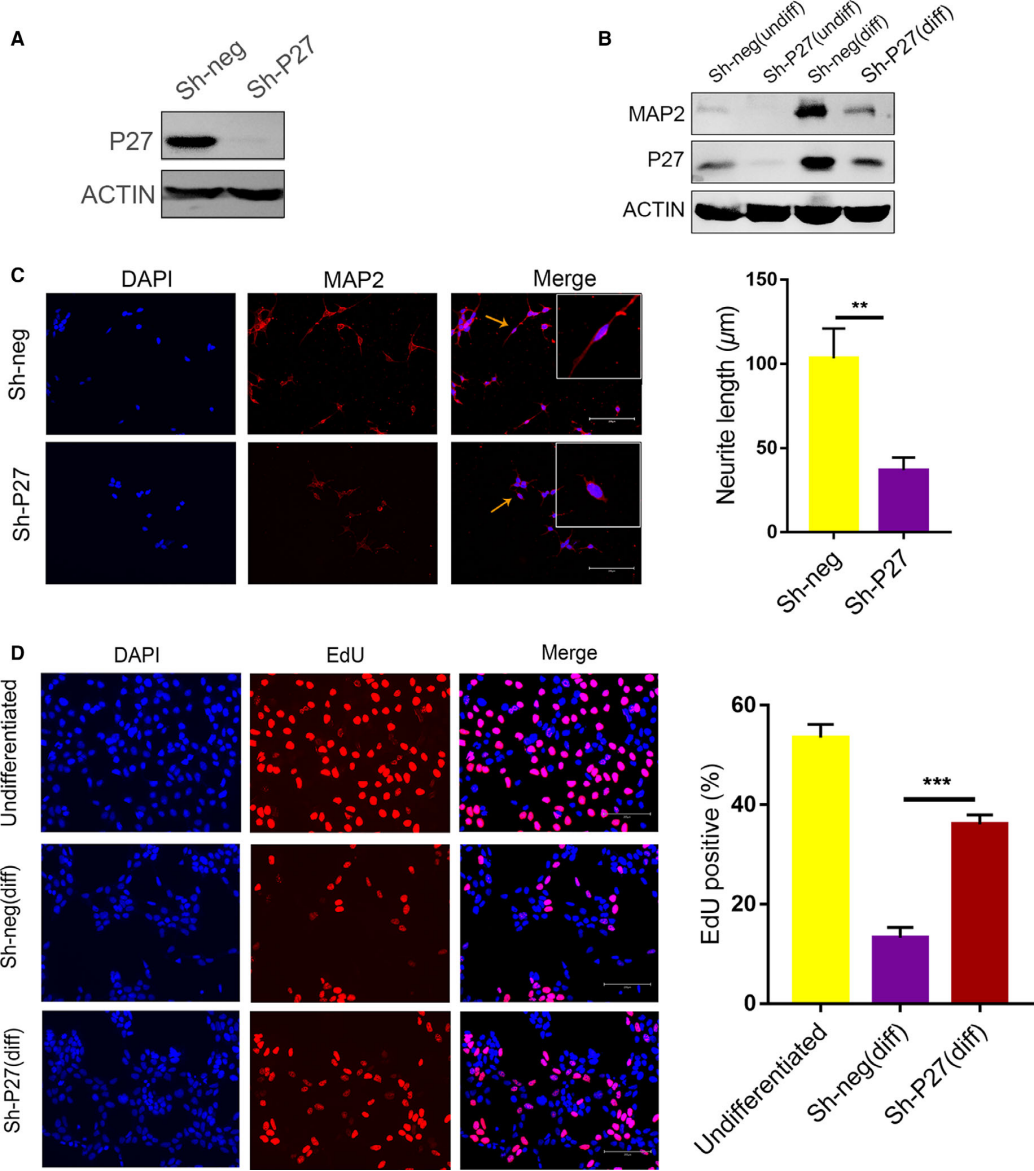
P27 knockdown inhibits neuronal differentiation and increases EdU‐positive cells of SH‐SY5Y cells. SH‐SY5Y cells were infected with sh‐P27 or control (sh‐Neg) lentiviruses. (A) Western blot analysis of the indicated protein expression in noninfected, control virus (sh‐neg), or sh‐P27 lentiviruses infected SH‐SY5Y cells, which were cultured in the presence of 10 μm RA for 4 days. (B) Western blot analysis of MAP2 protein expression in control (sh‐Neg), or sh‐P27 lentiviruses infected SH‐SY5Y cells, which were cultured in the absence or presence of RA for 8 days. (C) Immunostaining of neuronally differentiated SH‐SY5Y cells, which were cultured in the presence of 10 μm RA for 8 days, with an anti‐MAP2 antibody. Representative photomicrograph of MAP2‐positive cells. The neurite length was calculated in differentiated SH‐SY5Y cells (arrows: representative neurite). (D) SH‐SY5Y cells were aggregated in the presence of 10 μm RA for 4 days and incubated with EdU for 2 h before fixation. EdU‐positive cells were used for immunofluorescence analysis. The percentage of EdU‐positive cells was determined. Scale bars, 200 μm. Data are depicted as means ± SD of at least three independent experiments. **P < 0.01, and ***P < 0.001, as determined by the two‐tailed unpaired Student's *t*‐test (C) and one‐way ANOVA followed by the LSD *post hoc* test (D).

**Fig. 6:**
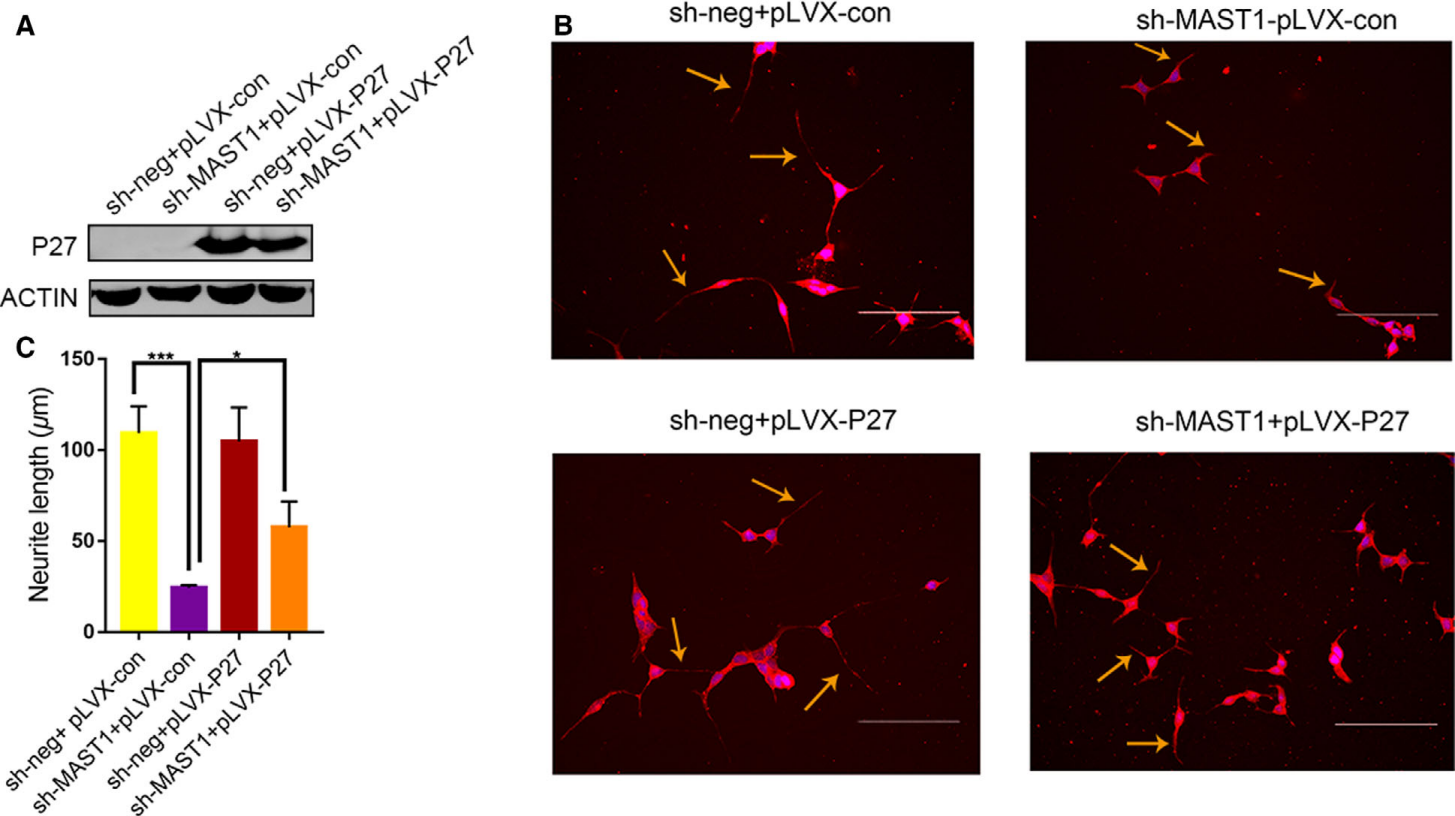
P27 re‐expression partially rescues the effect of MAST1 knockdown on SH‐SY5Y cell neuronal differentiation. P27 was inserted into the control (sh‐neg) and MAST1 shRNA (sh‐MAST1) constructs. (A) Western blot analysis of P27 protein expression in SH‐SY5Y cells. (B) Immunostaining of neuronally differentiated SH‐SY5Y cells, which were incubated in the presence of 10 μm RA for 8 days with an anti‐MAP2 antibody. (C) The neurite length was determined in differentiated SH‐SY5Y cells. Scale bars, 200 μm. Data are depicted as means ± SD of at least three independent experiments. *P < 0.05 and ***P < 0.001, as determined by one‐way ANOVA followed by the LSD *post hoc* test.

To investigate the possibility that P27 meditated MAST1 function in neuronal differentiation of RA‐induced SH‐SY5Y cells, we used lentiviral shRNA to knockdown P27 and detected the effects on neuronal differentiation. P27 shRNA virus (sh‐P27) significantly decreased the expression of P27 protein (Fig. 5A). The expression of MAP2 and neurite length was significantly decreased in P27 knockdown cells (Fig. 5B,C). There was also a significant increase in EdU‐positive cells in P27 knockdown cells compared with the sh‐neg control after RA treatment (Fig. 5D), indicating that both cell proliferation and neuronal differentiation were affected by depletion of P27, similar to depletion of MAST1. Furthermore, we examined whether P27 re‐expression could rescue neuronal differentiation induced by MAST1 silencing. Re‐expressing P27 in MAST1 knockdown cells caused an increase in neurite length compared with MAST1 knockdown cells (Fig. 6A–C). These results suggested that P27 is required for SH‐SY5Y cell neuronal differentiation and is a mediator of differentiation that is regulated by MAST1.

## Discussion

Neuronal differentiation plays an essential role in the formation of the central nervous system. Delineating the factors related to neuronal differentiation in cells is one of the most fundamental research topics in nervous system development. Here, we found that inhibiting MAST1 expression significantly restrained neuronal differentiation and inhibited cell cycle arrest by RA treatment. Meanwhile, downregulation of MAST1 inhibits P27 in SH‐SY5Y cells. Moreover, P27 re‐expression partially rescued neuronal differentiation in MAST1 knockdown cells and acted a meditator regulating the function of MAST1. Collectively, we have revealed that MAST1 is a critical intrinsic factor for neuronal differentiation in SH‐SY5Y cells.

The human *MAST1* gene is located in chromosome 19, which is one of the most gene‐rich chromosomes. In fact, MAST1 is considered to be a possible candidate gene responsible for the novel microdeletion syndrome of 19p13.13 [3]. In patients with 19p13.13 microdeletion, a region of overlap of approximately 311–340 kb containing 16 genes was identified [3]. NFIX, a gene located at 19p13.13, is also regarded as a candidate gene [3] for novel microdeletion syndrome of 19p13.13. In mouse model, Nfix was found to be essential for normal brain development [28], while loss of Nfix resulted in brain malformations, including partial agenesis of the corpus callosum and ventriculomegaly [29]. The CACNA1A gene, another gene located at 19p12.13, encodes the alpha‐1A subunit of calcium voltage‐gated channels, and mutations in this gene have been reported with episodic ataxia type 2 [30] and epilepsy [31]. However, the two genes did not completely explain the neurological phenotype of 19p13.13 microdeletion syndrome, indicating other factors contribute to the severity and variability of the phenotype. MAST1 is highly expressed in the central nervous system and is regulated during brain development [4], suggesting MAST1 has a potential role in neuronal development. In this study, the expression of MAST1 was determined to be significantly correlated with the degree of neuronal differentiation, with depletion of MAST1 significantly impairing neuronal differentiation, suggesting that MAST1 participates in the process and may have a role in 19p13.13 microdeletion syndrome.

What molecular mechanism underlies MAST1 in neuronal differentiation? Here, we showed that suppressing MAST1 destructed cell cycle arrest, suggesting that MAST1 might inhibit cell cycle progression, resulting in neuronal differentiation. Analysis of MAST1 knockdown SH‐SY5Y cells indicated that there was a dramatic reduction in the levels of P27 protein, and the pattern of P27 during SH‐SY5Y cell differentiation was similar to that of MAST1. High expression of P27, a cyclin‐dependent kinase inhibitor, in postmitotic neurons was associated with binding of CDK2, and blockage of its activity was involved in cell cycle arrest and was believed to promote neuronal differentiation [32]. We therefore speculated that P27 is a possible mediator of MAST1 function in SH‐SY5Y cell neuronal differentiation. P27 is regulated by various transcription factors (e.g., forkhead box O1) and pathways (e.g., E2F1‐topoIIβ signaling pathway), which are also important to neuronal differentiation [33, 34, 35]. The mechanism by which MAST1 regulates P27 expression is not clear and needs further investigation. A previous study showed that MAST1 stabilized PTEN [36] and that PTEN increases the levels of P27 [37]. Taken together, these observations support our speculation that P27 may function downstream of MAST1.

It is still unclear how neuronal differentiation progresses, but it is accepted that withdrawal from the cell cycle is a precondition for neuronal differentiation and that crosstalk between the signaling pathways that control the two processes is involved [11, 38]. In this study, we proposed that MAST1 was a novel intrinsic signal that was essential for neuronal differentiation in SH‐SY5Y cells. Our results revealed that MAST1 functions upstream of cell cycle regulatory genes to promote cell cycle exit and neuronal differentiation. Nevertheless, several limitations of this study should be noted. Exact mechanisms, such as whether p27 could be phosphorylated by MAST1 with a Ser/Thr kinase activity, should be further investigated.

To summarize, we demonstrated that MAST1 was induced during SH‐SY5Y, N2a, and P19 neuronal differentiation. We also showed the function of MAST1 in neuronal differentiation by demonstrating that MAST1 promoted neuronal differentiation by affecting cell cycle regulation. P27 accumulation during neuronal differentiation is associated with MAST1 expression, suggesting a possible mechanism by which MAST1 exerts its function. Finally, these results indicated that MAST1 may have a role in 19p13.13 microdeletion syndrome and could be a future therapeutic target in neuronal development disorders.

## Conflict of interest

The authors declare no conflict of interest.

## Author contributions

DM designed and conceived the study; TJ and JM done experiments and analyzed the data; TJ, HZ, and JM drafted this manuscript; DM, TJ, HZ, JM, JZ, and NJ revised the manuscript. And all authors read and approved the final manuscript.

## Supporting information


**Fig. S1.** Expression of the neuronal marker Tubb3 from different phases of SH‐SY5Y cell neuronal differentiation.
**Fig. S2.** Induced expression of MAST1 during N_2_a and P19 cell neuronal differentiation. (A) Western blot analysis of protein expressions of MAST1 and the neuronal markers Tubb3, from different phases of N_2_a cell neuronal differentiation. N_2_a cells which had cultured in the presence of 10 μM RA for 8 days. (B) Western blot analysis of protein expressions of MAST1 and the neuronal markers Tubb3 and Map2, from different phases of P19 cell neuronal differentiation. P19 cell aggregated in the presence of 1 μM RA for 4 days followed by plating on culture dishes for 6 days. Agg, aggregation; D, day.
**Fig. S3.** RNA interference targeting Mast1 impairs the neuronal differentiation of N_2_a cells. (A) Western blot analysis of Mast1 protein expressions in control (sh‐neg), and shMast1‐1, shMast1‐2 and shMast1‐3 lentiviruses infected N2a cells, which were cultured in the presence of RA for 4 days. (B) Immunostaining of neuronally differentiated N2a cells, which were cultured in the presence of RA for 8 days, with anti‐Tubb3 antibody. (C) The percentage of differentiated N2a cells was determined. Scale bars, 200 μm. Data are depicted as means ± SD of at least three independent experiments. **P < 0.01, as determined by the two‐tailed unpaired Student's *t* test.
