# Supplementary material for: MAST1 modulates neuronal differentiation and cell cycle exit via P27 in neuroblastoma cells
Source: FEBS Open Bio. 2020 Apr 29;10(6):1104–14. doi: 10.1002/2211-5463.12860 (PMC7262902; doi:10.1002/2211-5463.12860)
Supplement: Supplementary file 1 — Fig. S1. Expression of the neuronal marker Tubb3 from different phases of SH‐SY5Y cell neuronal differentiation. Fig. S2. Induced expression of MAST1 during N2a and P19 cell neuronal differentiation. (A) Western blot analysis of protein expressions of MAST1 and the neuronal markers Tubb3, from different phases of N2a cell neuronal differentiation. N2a cells which had cultured in the presence of 10 μM RA for 8 days. (B) Western blot analysis of protein expressions of MAST1 and the neuronal markers Tubb3 and Map2, from different phases of P19 cell neuronal differentiation. P19 cell aggregated in the presence of 1 μM RA for 4 days followed by plating on culture dishes for 6 days. Agg, aggregation; D, day. Fig. S3. RNA interference targeting Mast1 impairs the neuronal differentiation of N2a cells. (A) Western blot analysis of Mast1 protein expressions in control (sh‐neg), and shMast1‐1, shMast1‐2 and shMast1‐3 lentiviruses infected N2a cells, which were cultured in the presence of RA for 4 days. (B) Immunostaining of neuronally differentiated N2a cells, which were cultured in the presence of RA for 8 days, with anti‐Tubb3 antibody. (C) The percentage of differentiated N2a cells was determined. Scale bars, 200 μm. Data are depicted as means ± SD of at least three independent experiments. **P < 0.01, as determined by the two‐tailed unpaired Student's t test. [file FEB4-10-1104-s001.pdf]

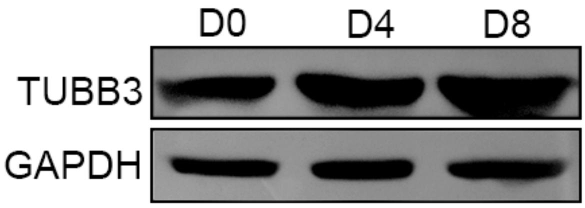

Supplementary Figure 1. Western blot analysis of protein expression of the neuronal marker TUBB3, from different phases of SH-SY5Y cell neuronal differentiation. D, day.

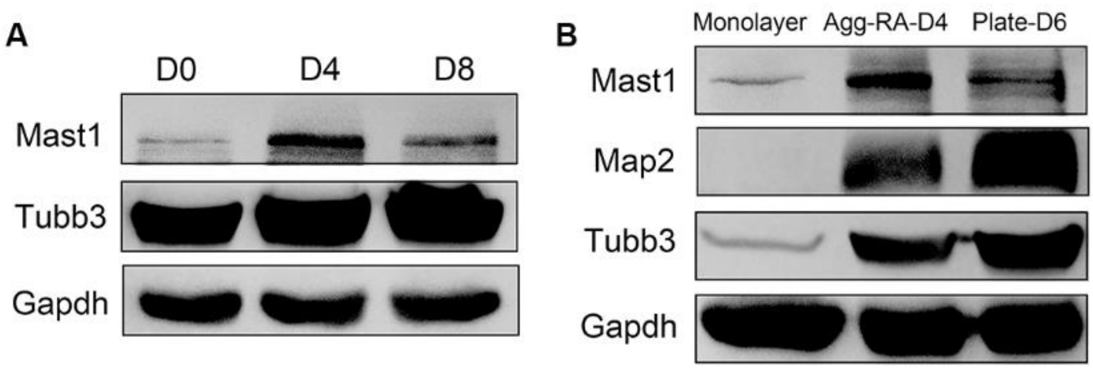

Supplementary Figure 2. Induced expression of MAST1 during N_2a and P19 cell neuronal differentiation. (A) Western blot analysis of protein expressions of MAST1 and the neuronal markers Tubb3, from different phases of N_2a cell neuronal differentiation. N_2a cells which had cultured in the presence of 10 μM RA for 8 days. (B) Western blot analysis of protein expressions of MAST1 and the neuronal markers Tubb3 and Map2, from different phases of P19 cell neuronal differentiation. P19 cell aggregated in the presence of 1 μM RA for 4 days followed by plating on culture dishes for 6 days. Agg, aggregation; D, day.

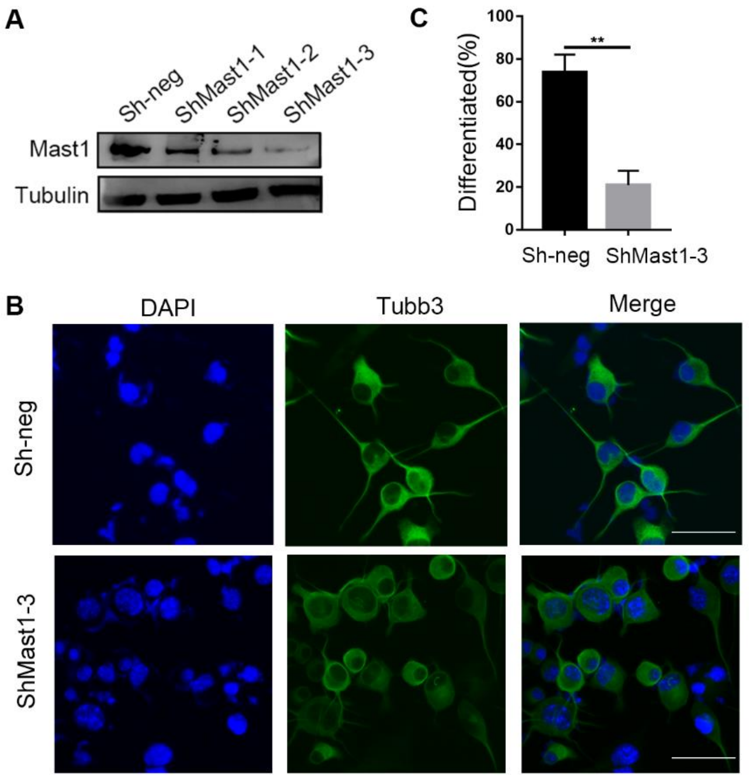

Supplementary Figure 3. RNA interference targeting Mast1 impairs the neuronal differentiation of N_2a cells. (A) Western blot analysis of Mast1 protein expressions in control (sh-neg), and shMast1-1, shMast1-2 and shMast1-3 lentiviruses infected N_2a cells, which were cultured in the presence of RA for 4 days. (B) Immunostaining of neuronally differentiated N_2a cells, which were cultured in the presence of RA for 8 days, with anti-Tubb3 antibody. (C) The percentage of differentiated N_2a cells was determined. Scale bars, 200 μm. Data are depicted as means ± SD of at least three independent experiments. **P<0.01, as determined by the two-tailed unpaired Student's *t* test.
